# Genetic Characterization of a Wheat Association Mapping Panel Relevant to Brazilian Breeding Using a High-Density Single Nucleotide Polymorphism Array

**DOI:** 10.1534/g3.120.401234

**Published:** 2020-04-29

**Authors:** Greg Mellers, Jorge González Aguilera, Nick Bird, Ana Lidia Variani Bonato, Sandro Bonow, Eduardo Caierão, Luciano Consoli, Flávio Martins Santana, James Simmonds, Andrew Steed, Gisele Abigail Montan Torres, Cristobal Uauy, Tally I. C. Wright, Pedro Luiz Scheeren, Paul Nicholson, James Cockram

**Affiliations:** * The John Bingham Laboratory, NIAB, 93 Lawrence Weaver Road, Cambridge, CB3 0LE, United Kingdom,; ^†^ EMBRAPA-Trigo, Caixa Postal 3081, 99050970, Passo Fundo, RS, Brazil, and; ^‡^ John Innes Centre, Norwich Research Park, Norwich, NR4 7UH, United Kingdom

**Keywords:** Single nucleotide polymorphism (SNP) genotyping, quantitative trait locus (QTL), genome-wide association scan (GWAS), genetic diversity, polyploid crop breeding, Fusarium head blight (FHB)

## Abstract

Bread wheat (*Triticum aestivum* L.) is one of the world’s most important crops. Maintaining wheat yield gains across all of its major production areas is a key target toward underpinning global food security. Brazil is a major wheat producer in South America, generating grain yields of around 6.8 million tons per year. Here, we establish and genotype a wheat association mapping resource relevant to contemporary Brazilian wheat breeding programs. The panel of 558 wheat accessions was genotyped using an Illumina iSelect 90,000 single nucleotide polymorphism array. Following quality control, the final data matrix consisted of 470 accessions and 22,475 polymorphic genetic markers (minor allele frequency ≥5%, missing data <5%). Principal component analysis identified distinct differences between materials bred predominantly for the northern Cerrado region, compared to those bred for southern Brazilian agricultural areas. We augmented the genotypic data with 26 functional Kompetitive Allele-Specific PCR (KASP) markers to identify the allelic combinations at genes with previously known effects on agronomically important traits in the panel. This highlighted breeding targets for immediate consideration – notably, increased *Fusarium* head blight resistance via the *Fhb1* locus. To demonstrate the panel’s likely future utility, genome-wide association scans for several phenotypic traits were undertaken. Significant (Bonferroni corrected *P* < 0.05) marker-trait associations were detected for *Fusarium* kernel damage (a proxy for type 2 *Fusarium* resistance), identifying previously known quantitative trait loci in the panel. This association mapping panel represents an important resource for Brazilian wheat breeding, allowing future genetic studies to analyze multiple agronomic traits within a single genetically diverse population.

Brazil is the second largest producer of wheat in South America, with a grain production of 6.8 million tons in 2016 (http://www.fao.org/faostat/en/#home). The majority of wheat cultivation in Brazil takes place in temperate climate zones of the southern states of Rio Grande do Sul and Paraná. However, wheat is also cultivated in the Cerrado climatic zone of Minas Gerais, Goiás, Mato Grosso and Mato Grosso do Sul in central Brazil. The development of new wheat varieties for cultivation in Brazil is undertaken by public institutes as well as private companies. Various studies have shown that the genetic gains in grain production arising from these breeding activities over the last 40 years has been between 1.2% (irrigated wheat, Cerrado region; Cargnin *et al.* 2008) and 2.9% (temperate region, Rio Grande do Sul; Follmann *et al.* 2017) per year. Such genetic gains have been achieved against a background of numerous biotic and abiotic pressures. Of particular note are two diseases of the wheat ear that are widespread in Brazil’s wheat producing regions: (A) *Fusarium* head blight (FHB), predominantly caused in southern Brazil by the fungus *Fusarium graminearum* Schw. (Teleomorph *Gibberella zeae* (Schw.) Petch.) (Astolfi *et al.* 2012), and (B) wheat blast, caused by the fungus *Magnaporthe oryzae* Triticum pathotype, which first emerged in the Brazilian state of Paraná in 1985 (Igarashi *et al.* 1986) and has since spread to other countries in both South America (Cruz and Valent 2017) and Asia (Malaker *et al.* 2016; Islam *et al.* 2016). Similarly, a suite of three abiotic pressures are generally acknowledged to be critical for Brazilian wheat production (Scheeren *et al.* 2008): (i) drought - a major limiting factor for rain-fed wheat cropping areas in the Cerrado, a region previously highlighted as a new frontier for increasing Brazilian wheat production (Poersch-Bortolon *et al.* 2016), (ii) increasing temperature - forecasted to reduce global wheat production by 6% for every degree Celsius increase in mean global temperature (Asseng *et al.* 2015), and (iii) aluminum toxicity - a widespread problem on the acidic soils of Brazil, where toxic aluminum ions inhibit root growth (De Sousa 2006), resulting in knock-on effects on crop growth and productivity, including an exacerbation of the effects of drought and heat stresses. Collectively, these principal biotic and abiotic factors represent key breeding targets for the protection of future Brazilian wheat yields.

Despite rapid advances over the last 40 years, there is evidence that genetic gains in Brazilian wheat yield are beginning to stagnate (*e.g.*, Cargnin *et al.* 2008), as is the case in many other countries around the world (Mackay *et al.* 2011; Brisson *et al.* 2010). Molecular and genetic characterization of the germplasm underpinning these gains in Brazilian grain yield could help breeding programs maintain, or even increase, such gains in the future. Indeed, the advent of association mapping techniques in cereal species has meant that it is possible to exploit both historic and *de novo* phenotypic data available for collections of both varieties and breeders’ lines to undertake quantitative trait locus (QTL) analyses via genome-wide association scans (GWAS) (Cockram and Mackay 2018), for example in wheat (Bentley *et al.* 2014) and barley (Cockram *et al.* 2010a). Such analyses rely on the availability of genome-wide genetic marker datasets, commonly consisting of thousands of single nucleotide polymorphism (SNP) markers, with various SNP arrays (*e.g.*, Cavanagh *et al.* 2013; Wang *et al.* 2014; Allen *et al.* 2017) and sequencing-based genotyping technologies (reviewed by Nguyen *et al.* 2019), recently having been applied in wheat. Previous GWAS studies have identified genetic loci controlling FHB (*e.g.*, Wang *et al.* 2017) and awning (*e.g.*, DeWitt *et al.* 2019) (for further examples, see also Discussion). As a complementary approach to the use of high-density genotyping approaches, the availability of genetic markers diagnostic or highly predictive of allelic state at genetic loci controlling major agronomic traits in wheat (*e.g.*, those listed on CerealsDB, https://www.cerealsdb.uk.net/cerealgenomics/, and subsequently published by Rasheed *et al.* 2016) allow known loci to be rapidly characterized in breeder-relevant germplasm, including association mapping panels (*e.g.*, Li *et al.* 2016). Despite the potential for such marker-informed approaches, relatively limited genome-wide genotypic investigation of Brazilian wheat germplasm has been undertaken to date (Schuster *et al.* 2009). Published studies investigating genetic diversity in Brazilian wheat have predominantly used relatively small numbers of varieties and genetic markers – for example, a study of 36 varieties with 23 simple sequence repeat (SSR) markers (Schuster *et al.* 2009). A notable exception is a recent study of 211 Brazilian varieties genotyped with an Axiom 30,000 feature WhtBrd-1 Array, and analyzed for genetic diversity (Scherlosky *et al.* 2018). However, no genetic association mapping panel specifically focused on use in Brazilian wheat breeding has been characterized both phenotypically and genotypically to date, nor has such a panel been characterized using functional genetic markers.

The aim of this study was to (1) assemble a wheat association mapping panel of relevance to Brazilian wheat breeding, (2) genotypically characterize the panel using a high-density SNP array, (3) augment the genome-wide SNP datasets via the addition of genotypic data for functional SNP markers, (4) demonstrate the utility of the association mapping panel by conducting GWAS for exemplar phenotypic traits, and (5) make these resources publicly available. To this end, we assembled a panel of 470 wheat varieties and breeders’ lines and genotyped it with a 90,000 feature SNP array. The panel was assessed for its genetic diversity, and the potential utility of the resource for quantitative trait locus (QTL) analysis demonstrated via preliminary genome wide association mapping of FHB resistance and awn presence/absence. To help further inform future exploitation of the panel for wheat breeding and genetic analyses a set of 26 functional SNP markers tagging known genetic loci were genotyped. Here, we make these germplasm, SNP and genetic resources publicly available, providing useful resources for wheat research and development in Brazil and beyond. The results are discussed in the context of current and future Brazilian wheat breeding targets.

## Materials And Methods

### Wheat germplasm

In total, 558 bread wheat accessions were collated from the collections maintained by Brazilian Agricultural Research Corporation (EMBRAPA)-Trigo, Brazil. The panel represents wheat germplasm released between 1852 and 2013, and was selected to be broadly representative of the genetic diversity used within Brazilian wheat breeding programs over the last 75 years at EMBRAPA-Trigo (Passo Fundo, Brazil). After quality control filtering, the panel consists of 470 accessions with 364 varieties and 64 breeders’ lines, as well as 42 synthetic lines (Supplementary Table 1), with 327 of the accessions of Brazilian origin.

### Field trials and phenotyping

Field trials for assessment of FHB resistance in the 2011, 2012 and 2013 seasons were undertaken at Embrapa-Trigo, Passo Fundo, Brazil (28°13’46” S, 52°24’07” W). Each genotype was planted, without replication, in a single plot consisting of 3 rows of 3 meters length, with 20 cm between rows. Following common protocols for assessment of FHB, the trial was divided into three sections according to the maturity group of each line: ‘early’, ‘medium’ and ‘late’ flowering, and entries randomized within each block. To aid FHB infection, plots were watered using mist irrigation for ten minutes six times per day (three times in the morning, and three times in the evening) in order to guarantee humidity during the whole night. Additionally, *Fusarium* infected wheat grain was spread between the rows of the trial 60 days after sowing. Percentage of *Fusarium* damaged kernels (%FDK), a proxy of disease spread within the ear (type 2 resistance), was assessed by sampling 1,000 seeds after harvest and manually identifying the proportion of *Fusarium* infected seeds. The number of genotypes was variable between years (Supplementary Table 2, intersection of accessions across years illustrated in Supplementary Figure 1) hence a between year analysis was performed. This took the best linear unbiased predictions (BLUPs) for genotypes in a design that was unbalanced across years from a model fitted to the FDK data with genotype and year as random effects, implemented using the package lme4 (Bates *et al.* 2015) in R, and subsequently termed the ‘meta-analysis’. The model used to fit phenotypic data across years was as follows:

$$y_{ij}=µ+G_{i}+T_{j}+e_{ij}{}_{}$$

where *y_ij_* is the %FDK of a plot, *µ* is the overall mean, *G_i_* and *T_j_* are random effects of genotype and year respectively, and *e_ij_* is the residual error of *y_ij_*. In the absence of weights for a generalized binomial mixed effect model, and due to the positive skew in the %FDK data, an initial transformation of the data were performed using box-cox transformation producing a lambda value of -1 for transformation. Final per genotype BLUPs were used as input for subsequent GWAS analyses. For the phenotype awn presence/absence, a binary assessment (1/0) of presence or absence was used as an input to subsequent analyses. Using Genstat (VSN International 2017a), generalized heritability (*H^2^*) was estimated on a line mean basis following Cullis *et al.* (2006):

$$H^{2}=\;1-\;\frac{V_{tt}}{(2\sigma_{G}^{2})\;}$$

where $\sigma_{G}^{2}$ was taken as the genetic variance and $V_{tt}$ was the average variance for difference between BLUPs (VSN International 2017b).

### DNA extraction and genotyping

Genomic DNA was extracted from leaf material harvested from a single individual per accession using previously described protocols (Palotta *et al.* 2003). DNA quality and quantity were assessed using agarose gel electrophoresis and Qubit (Thermo Fisher), respectively. DNA was diluted to a concentration of 60-200 ng/μl using PCR grade water (Sigma Aldrich) and submitted under subcontract to Bristol University or the Institute of Biological, Environmental and Rural Sciences (IBERS), Aberystwyth University for genotyping. Genotyping was performed using a 90k feature Illumina iSelect SNP array developed by Wang *et al.* (2014). SNPs were called using GenomeStudio v2011.1 (Illumina) using a no call threshold of 0.1. Clusters were called in the Polyploid genotyping package with a minimum of 15 individuals per cluster and a 0.07 cluster distance with DBSCAN. SNP loci with greater than four clusters were removed. All remaining SNP loci that could not be automatically clustered were manually curated. Of the 67,774 SNPs successfully assayed on the array, 22,625 were polymorphic and passed quality control thresholds of minor allele frequency (MAF) ≥5% and missing data <5%. Individual genotypes were then only kept with <5% missing data. Genetic markers were subsequently filtered again (<5% MAF), resulting in a total of 22,475 SNPs for downstream analyses (Supplementary Table 3). Inter-plate variation was assessed within GenomeStudio using replicate genotypes. Inter-machine variation between providers was visually assessed for fluorescence variance in GenomeStudio. Where required, missing data were imputed using softImpute v1.4 (Mazumder *et al.* 2010) in R v3.3.3. Markers were first centered and scaled with softImpute before imputing missing data. Additionally, a subset of 26 previously published KASP markers for genes that underpin economically important wheat traits (open access on CerealsDB and also as pubilshed by Rasheed *et al.* 2016) were genotyped using established protocols (Trick *et al.* 2012).

### Population structure analysis

All analyses were undertaken using the software, R v3.3.3 (R Core Team 2015). Of the 470 accessions, the synthetic hexaploid wheat accessions were removed due to their different origin, and the 22,625 genetic markers were re-filtered again on <5% MAF using a custom script. Principal component analysis (PCA) was conducted using the base package, ‘stats’, function ‘prcomp’. For PCA, the marker data were thinned using a custom script: where two markers had a pairwise correlation coefficient (*r*) > 0.75, only a single marker from the comparison was kept. PCA plots were generated using the R packages ggplot2 (Wickham 2016) and ggpubr (Kassambara 2019). Intra-chromosomal linkage disequilibrium (LD) between genetically mapped loci was calculated by the squared correlation coefficient (*r^2^*) using the function ‘LD.Measures’ implemented by the package LDcorSV (Desrousseaux *et al.* 2017) in R. Marker data from the 427 accessions were first thinned at a threshold of *r* < 1 using a custom script to remove co-located markers, leaving 8,527 markers with genetic map positions from the Wang *et al.* (2014) linkage map. Results were visualized per chromosome by plotting *r^2^* values against genetic distance and using a locally weighted scatterplot smoothing (LOWESS) to fit a line to the data, implemented by the ‘lowess’ function in R. For the A and B genomes, a smoothing span of 5% of data points was used, while for the D genome a smoothing span of 20% of data points was used.

### Genome wide association scans

GWAS was undertaken with the package, GAPIT (Lipka *et al.* 2012) using a Mixed Linear Model (MLM) algorithm as

y=β+Zμ+e

where *y* is the vector of observed phenotypes, *X* is the incidence matrix of fixed effects *β* is a vector of coefficients of the fixed effects (here the mean and one SNP), *Z* is the incidence matrix mapping for individual genetic effects, *μ* is the vector of individual genetic effects with Var(*μ*) = Kσ_g_^2^ where K is the kinship matrix estimated from the markers *e* is an *n* × *n* matrix of residual effect such that Var(*e*) = *I*σ_e_^2^. These analyses excluded the synthetic wheat lines due to their highly differentiated genetic structure. Correction for kinship was undertaken using the default kinship matrix constructed in GAPIT, from a subset of 4,313 high quality SNPs parsed from the full quality-controlled data matrix as follows: missing data <5%, MAF >5% and thinned by LD at *r* < 0.75 using a custom script. Skimming by *r* was undertaken to reduce the effect of uneven LD and SNP density on the calculation of kinship (Speed *et al.* 2012), an issue that is particularly acute in wheat. Missing data in the SNP set was imputed using iterative soft-threshold singular value decomposition, implemented in the R package, softImpute. Mapped SNPs published by Wang *et al.* (2014) were used for GWAS (6,215). Bonferroni-adjusted *p* values at a significance threshold of 0.05 or 0.01 were used for all genome wide association scans (*P* < 8.05x10^−06^ or 1.60x10^−06^, denoted by blue and red threshold lines, respectively). The wheat reference genome assembly (IWGSC RefSeq v1.0, cv. Chinese Spring. IWGSC, 2018), was used to determine the physical positions of significant (*p_adj_* < 0.05) markers of interest. SNP flanking DNA sequences were acquired from GrainGenes (Carollo *et al.* 2005. Available at https://wheat.pw.usda.gov/GG3/) and TriticeaeToolbox (Blake *et al.* 2016. Available at https://triticeaetoolbox.org/), and BLASTn (Altschul *et al.* 1990) was used to determine marker positions in the wheat physical map (e-value threshold < 1e^-20^).

### Data availability

SNP, KASP and phenotypic data are available in Supplementary Tables 1 through to 4, available at figshare. File Supplementary Table 1 contains details of the AM panel. File Supplementary Table 2 contains the phenotype data used for GWAS. File Supplementary Table 3 contains the 90k SNP genotypic data. File Supplementary Table 4 contains the KASP SNP data. Seed for the AM panel can be requested from Embrapa-Trigo: contact cnpt.chpd@embrapa.br. Supplemental material available at figshare: https://doi.org/10.25387/g3.12077124.

## Results

### Genome-wide genetic markers

After quality control, of the 67,774 SNPs successfully assayed on the array, 22,475 polymorphic SNPs remained for downstream analyses. All genetic markers were ordered using the wheat consensus genetic map (Wang *et al.* 2014), allowing 18,895 SNPs to be assigned a genetic map position (Supplementary Table 3). Analysis of SNP frequencies across the A, B and D sub-genomes of wheat found the B genome to possess the highest number of SNPs (9,716), while the D genome had the lowest (1,863). By chromosome, the number of SNPs ranged from 1789 (chromosome 5B) to 95 (4D). Markers were further thinned at a threshold of *r* < 0.95 to remove redundant SNPs in very high LD (as commonly applied in large genome temperate cereal crops, *e.g.*, Bentley *et al.* 2014), producing a thinned total of 8,721 SNPs for 470 individuals of which 6,215 had assigned genetic map positions (Wang *et al.* 2014).

### Population structure and linkage disequilibrium

**Figure 1:**
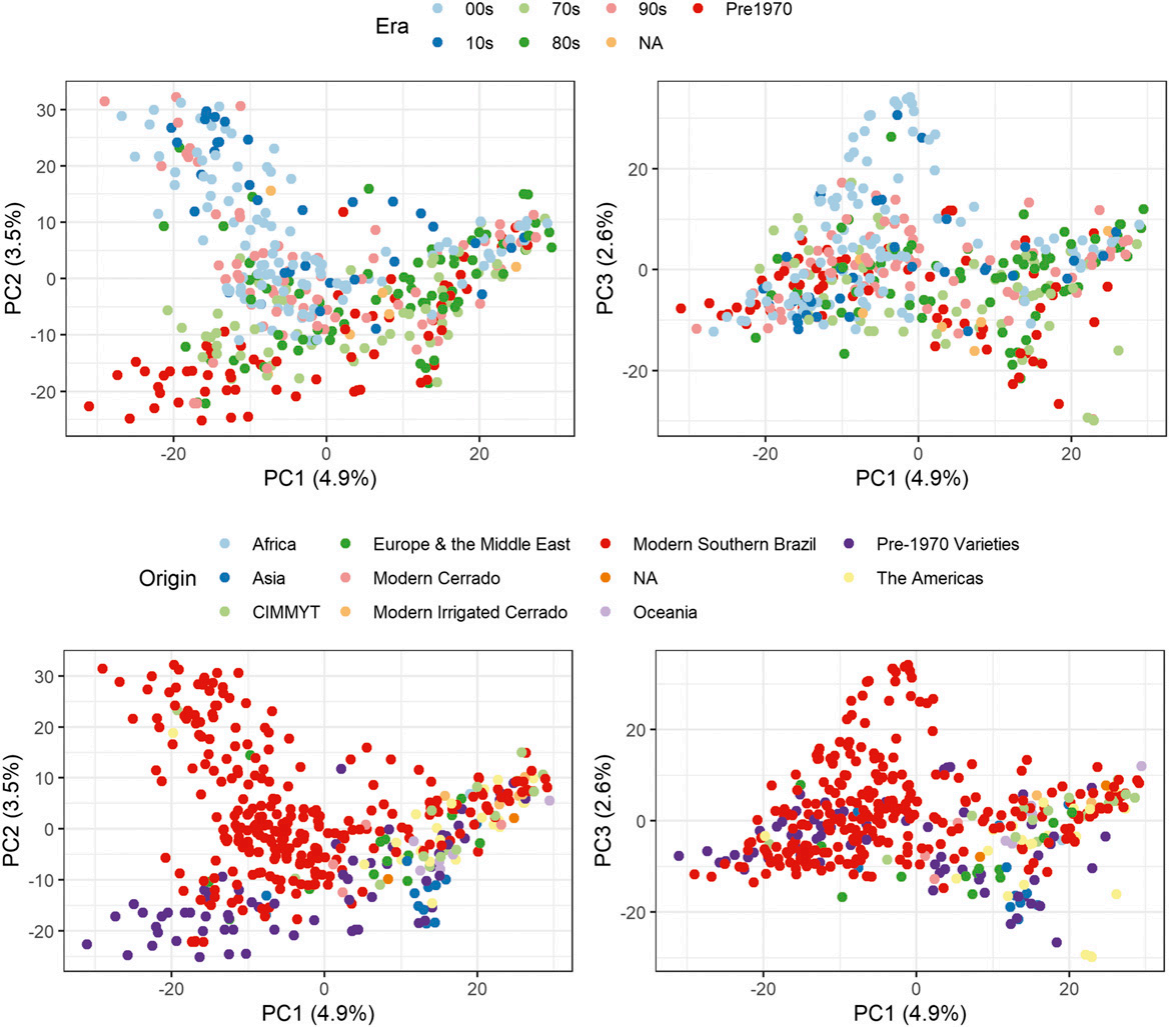
Principal component analysis of the genotyped association mapping panel (*n* accessions = 427) highlights major divisions in the material when considered by the era of variety release (top), and origin (bottom). Analysis of principle component 1 (PC1) *vs.* PC2 shows a division between the more modern germplasm released in the decades 2000, 2010 and those released before this time. Second, separate breeding targets for the northerly (modern Cerrado) and southerly wheat growing environments of Brazil result in segregation of material, with the more northerly material showing greater similarity to international material.

**Figure 2:**
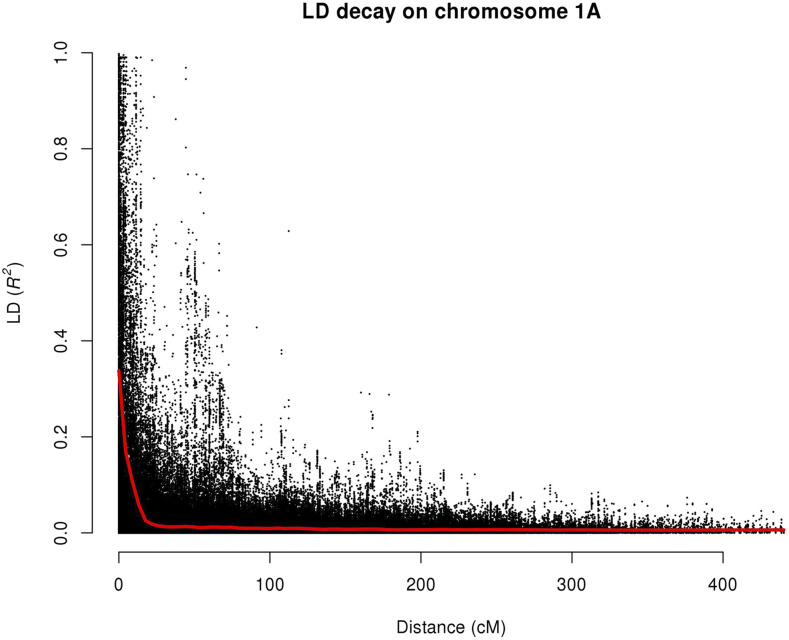
Intra-chromosomal linkage disequilibrium (LD) in the association mapping panel, as measured by *r^2^* between genetically mapped marker pairs. A LOWESS curve is fitted to the data, indicated in red. Wheat chromosome 1A is shown here, with LD plots for all other chromosomes provided in Supplementary Figure 2.

The geographic origin of the accessions of the association mapping panel was attributed to one of ten categories by the wheat breeders at EMBRAPA Trigo, Brazil including: Africa, the Americas, Asia, CIMMYT collections, Europe and the Middle East, southern Brazil, northern Brazil (*i.e.*, Cerrado region, irrigated and non-irrigated), Oceania, and pre-1970 (*i.e.*, before the Green Revolution). A subset of 4,313 SNPs parsed from the full Illumina iSelect dataset (missing data <5%, MAF ≥5% and thinned by LD at *r* < 0.75) was used to investigate the genetic substructure of the panel. Principal component (PC) analysis found the first three principal components to capture 11% of the total variance (Figure 1). Plotting PC1 against PC2 and overlaying information on era of variety release and geographic origin resolved some broad trends. The most striking was a central cluster of modern southern Brazilian lines released predominantly in the 2000s and 2010s largely separated from all other accessions via the PC2 axis. These modern materials were flanked on each side by a cluster predominantly represented by pre-1970s germplasm to one side, and a cluster which contained accessions from various origins on the other - including all of the accessions from the Brazilian Cerrado, and most of the international material, in particular all accessions from Asia, Oceania, the Americas and CIMMYT, as well as most of the remaining pre-1970s lines. The more modern material showed little clustering with year of release (Figure 1) but instead broadly grouped by region of cultivation in Brazil with southerly and northerly material predominantly clustering separately. Analysis of the kinship matrix generated using the same subset of 4,313 SNPs supports the broad groupings identified by principal component analysis (Supplementary Figure 2). Linkage disequilibrium for mapped markers was found to decay at a relatively slow rate, similar to that observed in previous wheat studies (*e.g.*, Maccaferri *et al.* 2015) (Figure 2; Supplementary Figure 3).

### Using diagnostic KASP markers to survey allelic variation for key agronomic traits

**Figure 3:**
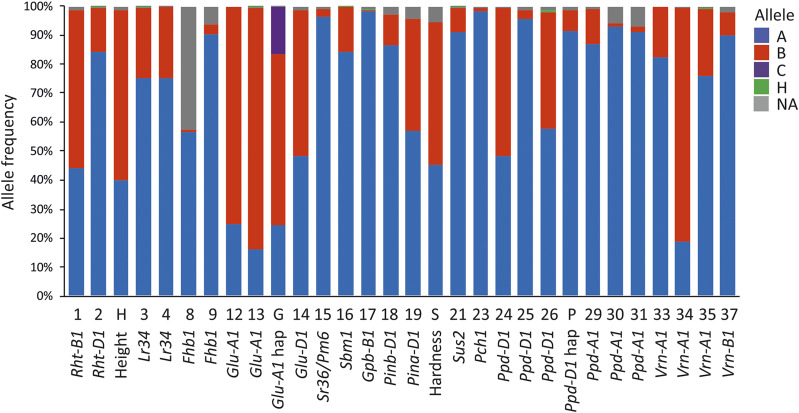
Summary of genotyping of 26 functional KASP markers in a subset of the association mapping panel (n = 349). KASP assay number is indicated on the x-axis, coded as 1 = wMAS000001, 2 = wMAS000002, etc. SNP allele calls are color coded: for disease resistance genetic loci, allele A (blue) represents the susceptible allele and allele B (red) the resistant allele. For all other SNPs, alleles A and B represent the wild type and mutant alleles, respectively. Allele NA = missing data/null allele call, allele H = heterozygous. Additionally, for some traits the genotypic results from a subset of KASP markers combine to predict overall phenotype, coded as: H = plant height (allele A = tall, B = semi-dwarf, surmised from KASP markers 1 and 2); G = *Glu-A1* allele (A = allele 1, B = allele 2*, C = allele null, surmised from KASP markers 12 and 13); S = seed hardness (A = soft, B = hard, surmised from KASP markers 18 and 19); *P* = *Ppd-D1* haplotype (A = photoperiod sensitive, B = photoperiod insensitive, surmised from KASP markers 24-26).

Twenty-six published KASP markers diagnostic for allelic state, or closely linked to previously identified loci (*i.e.*, predictive of allelic state) controlling a range of agronomically relevant traits were used to genotype 349 accessions of the AM panel (Figure 3. Supplementary Table 4). Using diagnostic markers for allelic state at the two major loci controlling plant height (*RHT-B1* and *RHT-D1*; KASP markers wMAS000001 and wMAS000002, respectively), 59% of accessions were classified as being ‘semi-dwarf’ in stature, while the remaining lines that lacked semi-dwarfing alleles at either of the two loci were classified as ‘tall’. Mutant *Rht-B1b* or *Rht-D1b* alleles were responsible for the semi-dwarf phenotype in 75% and 25% of the accessions carrying dwarfing alleles, respectively. Genotyping ten markers assessing eight disease resistance loci found resistance alleles to be present at relatively low frequency, with eye spot resistance alleles conferred by *Pch1* representing the most infrequent (1%; wMAS000023). Resistance alleles for *Fusarium* head blight conferred by the major resistance locus *Fhb1* were predicted in 3% of lines (wMAS000009, wMAS000009). Resistance alleles to stem rust conferred by *Stem rust 36* (*Sr36*) were predicted to be present at 3% (wMAS000015). The most frequent resistance alleles were identified for the soil borne mosaic virus resistance locus *Sbm1* (15%; wMAS000016) and the yellow rust resistance gene *Lr34* (25%; wMAS000003 and wMAS000004). Four loci controlling grain quality traits were assessed: (1) via the two-locus haplotypes derived from the assays targeting the *Pina-D1* and *Pinb-D1* loci (wMAS000018 and wMAS000019), 55% accessions were classified as having ‘hard’ grain (allele combination *Pina-D1b* + *Pinb-D1b*) and 45% as ‘soft’ grain (*Pina-D1a* + *Pinb-D1b* or *Pina-D1b* + *Pinb-D1a*). (2) Alleles contributing to the composition of high molecular weight glutenin subunits (HMW-GS) in the grain were predicted via assays for the *Glu-A1* (wMAS000012, wMAS000013) and *Glu-D1* (wMAS000014) loci. The two-SNP haplotypes generated allowed classification of *Glu-A1* alleles as ‘null’ (16%), ‘1Ax1’ (24% of lines assayed) or ‘1Ax2*’ (59%), ordered here in increasing positive effect on bread making quality. Genotyping of the SNP linked to *Glu-D1* predicted 51% of lines to be classified as ‘5+10’, indicative of improved bread making quality, while the remaining 49% of lines were predicted to carry allele(s) for the ‘2+12 and others’ gluten class associated with weaker gluten. (3) Via assessment of the KASP marker assaying a SNP linked to the *Gpc-B1* locus (wMAS000017), the allele conferring high grain protein content was predicted to be present in 1% of accessions. (4) Assessment of allelic state at the sucrose synthase *Sus2-2B* locus (wMAS000021) found 8% of lines to carry the T586C_hapH allele associated with higher thousand kernel weight. Finally, four major flowering time loci were assayed for a subset of known alleles using ten KASP markers: the photoperiod response loci *PPD-D1* and *PPD-A1*, and the vernalization response loci *VRN-A1* and *VRN-B1*. At *PPD-D1*, assessment for the presence of the 2,089 bp promoter deletion first characterized in the variety Ciano67 identified photoperiod insensitive alleles in 48% of lines (wMAS000024). The photosensitive insensitive allele conferred by the insertion of a Mariner transposable element within *PPD-D1* intron-1 was identified at low frequency (3%; wMAS000025) in the lines assayed. The *PPD-D1* 5 bp exon 7 deletion conferring photoperiod insensitivity was identified in 40% of lines (wMAS000026). Collectively, *PPD-D1* photoperiod insensitive alleles were present in 91% of the accessions screened. Allelic variation at the T (‘winter’ allele) to C (‘spring’ allele) SNP in exon-4 of the *AP1*-like gene underlying the *VRN-A1* locus found 19% of the accessions tested to carry ‘spring’ alleles (wMAS000034), while the assay targeting the ‘spring’ *Vrn-A1a* allele conferred by a deletion within the promoter was present in 23% of lines (wMAS000035). Based on these two assays, a total of 42% of lines were predicted to carry ‘spring’ alleles at *VRN-A1*. The SNP previously reported to be associated with ‘spring’ and ‘winter’ alleles at *VRN-A1* (Yan *et al.* 2004) and subsequently converted to KASP marker wMAS000033 found 17% of lines to carry ‘spring’ alleles. Finally, genotyping the *VRN-B1* locus found ‘spring’ *Vrn-B1b* alleles to be present in 8% of lines (wMAS000037).

### Genome wide association scans

**Figure 4:**
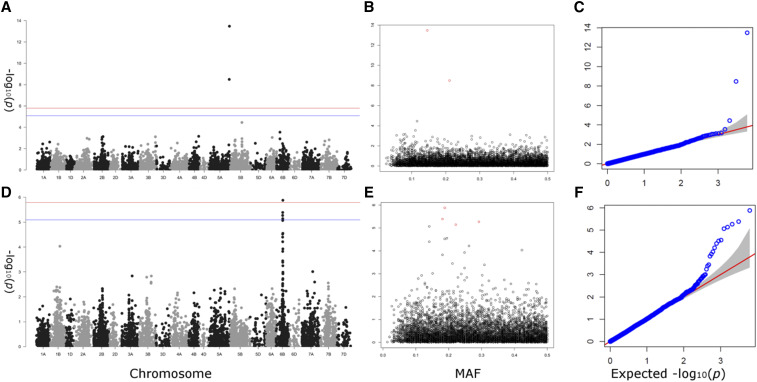
Genome wide association scans for the awn presence/absence (A, B, C) and % *Fusarium* damaged kernels (D, E, F). Manhattan plots of kinship corrected association analyses (A, D), minor allele frequency (MAF) against significance (B, E) and observed-expected significance (C, F). The potentially higher number of structurally driven significant loci (*P* < 0.05) in % *Fusarium* damaged kernels likely derive from the fewer lines (n = 149) in this analysis. However, the highly significant (*P* < 0.01) region is consistent with the previously mapped region.

To validate this panel for future use toward genetic analysis of traits relevant to Brazilian wheat production, phenotypic data were collected for two traits for subsequent GWAS. A subset of the full panel (n = 149) was phenotyped for %FDK in 2011, 2012 and 2013, as well as for awn presence/absence (n = 199) (Supplementary Table 2). For %FDK a meta-analysis was performed between years for GWAS. The overall mean of the resulting BLUPs for %FDK was 5.46 with a standard deviation of 0.69 and the data followed a normal distribution (Supplementary Table 2). For the qualitative awn trait, the number of genotypes with and without awns was 175 and 24, respectively. Of the traits studied, both showed significant marker trait associations (Figure 4). For awns presence/absence (n = 199) two markers on chromosome 5A were found to be highly significant (p_adj_ < 0.01), BobWhite_c8266_227 at 703.91cM and RAC875_c8642_231 at 709.71cM. For the meta-analysis of %FDK, which had a generalized heritability of 0.68, four significant markers (p_adj_ < 0.05) were identified on chromosome 6B, between 218.86 and 226.64 cM (Table 1).

**Table 1:** Details of significant (*p_adj_* <0.05) single nucleotide polymorphisms (SNPs) identified by genome-wide association scans (GWAS) for awn presence/absence and resistance to *Fusarium* disease, as assessed via percentage of *Fusarium* damaged kernels (FDK). ^†^Wang *et al.* (2014). ^‡^IWGSC Refseq v1.0 (IWGSC, 2018). Chr = chromosome. PVE = percentage variation explained. N = number of lines. MAF = minor allele frequency

Trait	Marker	Chr^†^	Genetic pos (cM)^†^	Physical pos (Mb)^‡^	PVE	*p*-value	MAF
Awn	BobWhite_c8266_227	5A	703.91	698.51	8.5	3.23 x 10^−9^	0.21
Awn	RAC875_c8642_231	5A	709.71	509.60	14.8	3.34 x 10^−14^	0.15
FDK	Kukri_c25377_240	6B	218.86	174.57	11.4	5.37 x 10^−6^	0.19
FDK	IAAV2161	6B	221.38	408.24	11.7	4.08 x 10^−6^	0.18
FDK	Excalibur_c23462_677	6B	222.92	438.93	11.1	7.23 x 10^−6^	0.22
FDK	BS00103275_51	6B	226.64	453.93	13.0	1.31 x 10^−6^	0.18

## Discussion

The analyses in this study centered on wheat germplasm used in Brazilian breeding programs since 1935. These genotypes represent the diversity of lines employed in breeding at Embrapa, and include varieties released for cultivation across all Brazilian wheat growing regions. In addition, lines were also included from global germplasm that had specific ongoing utility to Brazilian germplasm, or which may have relevance in the near future. Our aim was to provide a comprehensive survey of genetic variation of relevance to the Brazilian wheat gene pool, understand how this total variability is distributed in the more recently developed genotypes, and to demonstrate the utility of the resources generated for genetic analysis of agronomic traits.

### Population structure

The broad trend toward a three-cluster separation shown by PCA was supported by the results of the kinship matrix (Figure 1; Supplementary Figure 2). These clusters can be broadly grouped according to the age and geographic origin of the accessions, with modern Brazilian germplasm from southern regions and the northern Cerrado region predominantly grouping within largely separate PCA clusters - although some mixing is evident of southern germplasm within the PCA cloud containing the majority of the northern Brazil germplasm. The large environmental differences between the Cerrado and Southern Brazilian agricultural environments necessitate largely independent breeding priorities, and our analyses of population structure indicate this may have led to the genetic differentiation of varieties bred for cultivation in these two regions. Wheat production in the Cerrado is typified by low yield potential when irrigation is not used, and with relatively high yield potential when irrigated (>8 tons per ha). A lack of *Fusarium* disease prevalence means that a broad range of international material, particularly lines adapted to drought environments and CIMMYT material, is suitable for breeding for improved yield in this more northerly Brazilian environment. In contrast, the major *Fusarium* disease pressure experienced in the southern Brazilian wheat growing regions have thus far limited the use of some international material for breeding of varieties targeting the more temperate climates. The potential long term effects of breeding for these two distinct agricultural environments is likely reflected in the PCA (Figure 1), in which material bred for southerly cultivation sits broadly independent of most other material in this study; including both the germplasm from international sources as well as the breeding material of the Cerrado region.

### Genetic control of *Fusarium* disease resistance

Meta-analysis of three years of field data on the percentage of *Fusarium* damaged kernels found the generalized heritability of the trait to be 0.68. The peak SNPs identified by GWAS were located on chromosome 6B. Previous work in wheat has identified several major QTL, including two well defined regions for *Fusarium* resistance (Buerstmayr *et al.* 2009). The first of these, *Fusarium head blight 1* (*Fhb1*) on chromosome 3B, controls resistance predominantly through limiting the spread of disease through the rachis from an infected floret (type 2 resistance) via the action of a pore-forming toxin-like (PFT) gene (Rawat *et al.* 2016). The second, *Fhb2* on 6B, has been suggested to reinforce the cell wall and sequester deoxynivalenol (DON) toxins reducing pathogen spread within the ear (Dhokane *et al.* 2016). The peak markers identified in this Brazilian association mapping panel (covering a region of 279.36 Mb) (Table 1) span the chromosome 6B region previously identified via fine-mapping of *Fhb2* (Cuthbert *et al.* 2007) which spans a region of 102.3 Mb (anchored between 217.96 to 320.26 Mb on 6B by markers gwm644 and gwm133, respectively). This result agrees with previous data, where spread of the disease within the ear was found to be regulated by *Fhb2* (Buerstmayr *et al.* 2009), with resistance alleles at this locus reducing percentage of FDK. Indeed, a QTL conferring resistance to both *Fusarium* disease and FDK identified via marker gwm88 has previously been reported to be located to the *Fhb2* locus using a biparental mapping population constructed from the Brazilian varieties Frontana and Mini Manó, which show moderate and low resistance to *Fusarium*, respectively (Ánges *et al.* 2014). BLASTn analysis finds gwm88 to be located on chromosome 6B at 430.08 Mb, close to the location of the most significant SNP identified here by GWAS (BS00103275_51, 453.93 Mb). Hence, the association mapping panel genotyped here is sufficient with low numbers of phenotyped accessions (n = 149) to identify loci relevant to *Fusarium* disease resistance, and shows that existing Brazilian germplasm, such as the variety Trigo-BR24, potentially carry type 2 resistance alleles at *Fhb2*. GWAS for FHB resistance has been previously conducted using various germplasm sources, including accessions predominantly from the USA (Arrunda *et al.* 2016; Liu *et al.* 2019; Tessmann *et al.* 2019; Wang *et al.* 2017), Europe (Kollers *et al.* 2013; Miedaner *et al.* 2011) and Asia (Li *et al.* 2016; Wu *et al.* 2019), as well as panels derived from accessions around the world (Hu *et al.* 2020). While *Fhb2* has previously been reported using bi-parental populations, our work is the first time that *Fhb2* has been identified by GWAS as far as we are aware. The information on which FHB resistance genes are present, or absent, in the association mapping panel used in our study will inform future Brazilian wheat breeding approaches. The ability to identify QTL by GWAS depends on various factors, including marker allele frequencies in the panel. Thus, while the GWAS undertaken for FHB and awning demonstrates the general utility of the panel for use in GWAS, successful QTL identification for other phenotypes will of course depend on a trait-by-trait and QTL-by-QTL basis. Indeed, we note that the inability to detect *Fhb1* via GWAS in our panel was likely due to the low frequency of resistance alleles at this locus in the association mapping panel, which was below the 5% MAF threshold for inclusion of SNPs in the association scans. As an infection of the wheat ear, FHB directly impacts grain yield (*e.g.*, Schuman and D’Arcy 2006; Zhang *et al.* 2011a; Reis & Carmona 2013). Additionally, FHB affects grain quality due to the accumulation of mycotoxins, predominantly DON. As these mycotoxins are harmful to humans and animals, grain contamination above threshold levels has a direct impact on grain use and trade. Future work in this association mapping panel to extend the available phenotypic data should allow for the identification of additional *Fusarium* resistance loci in the future. Combined with using marker-assisted tracking of novel and known *Fusarium* resistance loci (such as *Fhb1*, for which we find resistance alleles to only very rarely be present in the panel, <3%), this has the potential to enhance breeding of future Brazilian wheat varieties with enhanced disease resistance and grain quality. A locus conferring type 1 FHB susceptibility has previously been reported closely linked to the semi-dwarf *Rht-D1b* allele on chromosome 4D (Srinivasachary *et al.* 2008). While semi-dwarfing alleles were found to be more commonly conferred by *Rht-B1b* alleles in the panel, the *Rht-D1b* semi-dwarfing allele was nevertheless observed at a frequency of 16%. GWAS did not detect a susceptibility locus linked to *RHT-D1*, and further investigation would be needed to clarify the allelic status of the 4D FHB susceptibility locus in this panel.

### Genetic control of awning

GWAS for awning identified a major genetic locus on the long arm of chromosome 5A at approximately 704 cM. In wheat, three dominant inhibitors of awning have previously been identified: *Hd* (Hooded, chromosome 4A), *B1* (*Tipped 1*, 5A) and *B2* (*Tipped 2*, 6B) (McIntosh *et al.* 1998; Kato *et al.* 1998, Yoshioka *et al.* 2017). While *Hd* and *B2* remain only coarsely mapped, the *B1* locus has previously been mapped to a 7.5 cM interval on the long arm of chromosome 5A, distal to the 4AL/5AL translocation breakpoint (Mackay *et al.* 2014), which was further fine mapped to a 2.2 cM interval (Yoshioka *et al.* 2017). More recently, a C2H2 zinc finger transcription factor has been identified as a candidate gene for *B1* using GWAS in an association mapping panel and by mapping in a biparental populations (DeWitt *et al.* 2019; Wang *et al.* 2019), located at 698.529 Mb in the reference wheat genome sequence assembly. The significant SNP for awning identified here by GWAS (BobWhite_c8266_227, 698.508 Mb, Table 1) is located ∼21 kb from the C2H2 zinc finger candidate gene, and has previously been identified as the most predictive marker for awning in a UK Multiparent Advanced Generation Inter-Cross (MAGIC) population (Mackay *et al.* 2014).

Inspection of the reference genome assembly and gene model annotations for cv. Chinese Spring (IWGSC, 2018) shows that the fine-mapped *B1* region (690.31 - 698.19 Mb, anchored via markers WABM232824 and WMS291, respectively) (Yoshioka *et al.* 2017) also contains the previously identified genes underlying the cereal *VRN-2* vernalisation locus (*ZCCT1*, ∼698.18 Mb on chromosome 5A) (Yan *et al.* 2004, Cockram *et al.* 2010b) which control flowering time and seasonal growth habit in response to low temperatures (termed ‘vernalization’). This indicates it could be of interest to explore whether the preference for awned varieties in warmer climates might also be due to linkage to specific seasonal growth habit alleles at *VRN-2*. By anchoring SNPs identified here as highly significant to the wheat reference genome, as well as those previously shown to have significant association with awn length on chromosome 5A (Yoshioka *et al.* 2017), we find the physical region identified by GWAS here (covering a region of 188.91 Mb. Table 1) spans the fine-mapped 5A region previously identified by Yoshioka *et al.* (2017), demonstrating the association mapping panel assembled in this work is sufficient with limited awn phenotype data (n = 199) to identify target genetic regions.

### KASP markers for the remaining disease traits

*Lr34* encodes an ABC-type transporter and confers resistance to a range of wheat fungal pathogens: *Puccinia triticina*, *P. striiformis* f. sp. *tritici*, *P. graminis* f. sp. *tritici* and Blumeria graminis (Krattinger *et al.* 2009). While stripe rust is not a particularly common pathogen of wheat in Brazil, several novel adult plant resistance loci have recently been identified in Brazilian wheat variety Toropi, as well as the multi-rust resistance locus Lr46/Yr29 (Rosa *et al.* 2019). We found resistant alleles at Lr34 to be relatively common in the AM panel, present in around a quarter of the lines, with KASP genotyping confirming the absence of Lr34 resistance alleles in the variety Toropi. Resistance to the related fungal wheat pathogen *Puccinia graminis* sp. *tritici*, the causal agent of stem rust, is conferred by multiple genes including *Sr2* and *Sr36*. Both of these resistance genes originated from bread wheat relatives: *Sr2* from *T. turgidum* var. *dicoccum* cv. Yaroslav and *Sr36* from *T. timopheevi*. While stem rust occurs in Brazil, major epidemics are currently rare, despite reports of increased cultivation of susceptible varieties in Southern America (Germán *et al.* 2006). The low incidence and severity of stem rust in Brazil is mirrored here by the predicted low occurrence of resistance alleles at the two loci assayed, particularly *Sr36*, present in just four Brazilian varieties. Soilborne wheat mosaic virus (SBWMV) is mediated via the soil borne fungus *Polymyxa graminis* Led. with losses of up to 50% reported in infected wheat fields in southern Brazil (Caetano 1982). Despite this, the SBWMV resistance locus *Sbm1* (Bass *et al.* 2006) was predicted here to be present in 15% of the lines surveyed. A second locus, *Sbm2*, has also been reported to confer increased resistance to SBWMV, although only when present in combination with resistant alleles at *Sbm1* (Bayles *et al.* 2007). Using a SNP linked to *Sbm1*, The Brazilian variety Embrapa 16 is known to carry SBCMV resistance, mediated by two genetic loci (Barbosa *et al.* 2001). However, genotyping with the *Sbm1* linked KASP marker predicted Embrapa 16 not to carry a *Sbm1* resistance allele. Therefore, either this variety carries previously uncharacterized SBWMV resistance loci, the *Sbm1* linked marker is not robust in wider germplasm pools, or variation exists at *Sbm1* in accessions of Embrapa 16 held at different genebanks. For eyespot, as this disease is not endemic to Brazil, resistance conferred by *Pch1* on the distal end of chromosome 7D is not a direct breeding target. *Pch1* resistance originated from the wheat relative *Aegilops ventricosa*. As this alien introgression is also associated with linkage drag for undesirable alleles (Pasquariello *et al.* 2017), it is useful to determine allelic status at *Pch1* in order to select against the underlying alien introgression within a Brazilian breeding context. Here, KASP genotyping predicts the *Pch1* resistance allele to be present in just four accessions, indicating the associated alien introgression does not currently present a significant source of potentially detrimental variation in the germplasm assessed here.

### KASP markers for end-use quality traits

Wheat end-use quality is a key target for breeders and growers, with traits such as dough gluten content and grain hardness determining grain sale price and the products that the grain can be used for. Gluten content is determined by a class of proteins known as the high molecular weight glutenin subunits (HMW-GS), the low molecular weight glutenin subunits (LMW-GS) and the gliadins. Alleles at several genetic loci, including *Glu-A1* and *Glu-D1*, are known to control variation for HMW-GS (*e.g.*, Zhang *et al.* 2011b). Here, of the lines carrying ‘null’ low glutenin quality alleles at *Glu-A1*, 74% also carried the SNP indicative of low glutenin quality alleles at *Glu-D1*, indicating selection for end-use for biscuit making or animal feed. For lines carrying the high glutenin quality ‘1Ax2’ *Glu-A1* allele, the approximately 50:50 split of high *vs.* low quality alleles at *Glu-D1* highlights the balance of glutenin subunits selected during breeding for the specific end-use quality desired. Grain endosperm hardness has a major impact on milling properties and is largely conferred by the puroindoline (*Pina* and *Pinb*) genes, with mutation in one or both genes resulting in hard grain. While differences in *Pin* gene expression has also been associated with grain hardness (Nirmal *et al.* 2016), here the two KASP markers employed assayed for wild type (*Pina-D1a*, *Pinb-D1a*) or mutant *Pina-D1b/Pinb-D1b* alleles, previously reported to be the most frequently observed mutant alleles in worldwide wheat collections (Lillemo *et al.* 2006). The predominance of grain hardness conferred by the *Pinb-D1b* allele in the AM panel (79% of all hard lines) mirrors previous observations in which 86% of hard wheat lines from the CIMMYT breeding program were found to carry the *Pinb-D1b* allele (Lillemo *et al.* 2006).

### KASP markers for phenology traits

Homeologous genes controlling flowering time in response to vernalization (*VERNALIZATION-A1* [*VRN-A1*], *VRN-B1*, *VRN-D1*) and photoperiod (*PHOTOPERIOD-A1* [*PPD-A1*], *PPD-B1*, *PPD-D1*) have a major influence on wheat flowering time, and thus adaptation and grain yield. Recessive wild-type *vrn-A1*, *vrn-B1* and *vrn-D1* alleles confer the vernalization responsive phenotype. Mutations in the promotors, introns and exons of *VRN-1* are known to result in the loss or reduction of the vernalization requirement (reviewed by Bentley *et al.* 2013). Collectively, markers within the *VRN-1* genes diagnostic for the ‘spring’ alleles *Vrn-B1b* (Díaz *et al.* 2012), *Vrn-A1a* (Yan *et al.* 2004) and the ‘2147-type’ exon-IV mutation (Díaz *et al.* 2012), as well as a linked SNP for a third spring *Vrn-A1* allele, predicted 57% of varieties to be spring, with no lines found to carry spring *Vrn-A1* and *Vrn-B1* alleles according to the polymorphisms assayed. However, it should be noted the KASP assay for allelic variation at the *VRN-D1* locus failed to provide reliable allele calls, and so was excluded. As mutations at *VRN-D1* are reported to have the strongest ‘spring’ alleles, it is likely that many of the remaining lines would be found to be genetically spring-type if genotyped for allelic state at *VRN-D1*. Considering all three assays for *PPD-D1*, almost all lines investigated (93%) were identified as carrying photoperiod insensitive alleles. This is as expected, as in Brazil wheat is typically grown as part of a cycle of three crop rotations per year, suiting rapidly maturing photoperiod insensitive lines. Of those lines found to lack insensitive alleles at *PPD-D1*, five possessed insensitive alleles at *PPD-A1*. These were all synthetic wheat lines originating from the wheat breeding institute CIMMYT, and carried the 1,117 bp promotor deletion first identified in line GS-105 by Beales *et al.* (2007). The predominance of this photoperiod insensitive allele in the synthetic lines screened indicates it was most likely brought into the bread wheat breeding pool via tetraploid wheat accessions used at CIMMYT during the creation of synthetic wheat lines.

### Conclusions

Characterization of genetic variation can increase the efficiency of breeding programs, helping selection of lines that maximize genetic diversity, and the introduction of specific traits and characteristics beneficial to regional agricultural environments. The wheat association mapping panel assembled here is the largest and best characterized of its kind specifically aimed to be relevant to Brazilian wheat breeding. We provide detailed genotypic datasets and analyses using the panel and demonstrate the utility of the resources generated by identifying significant marker-trait association via GWAS. Collectively, the resources generated here will help underpin future breeding programs, and aid future genetic analysis of agronomic traits of importance to Brazilian agricultural environments.
